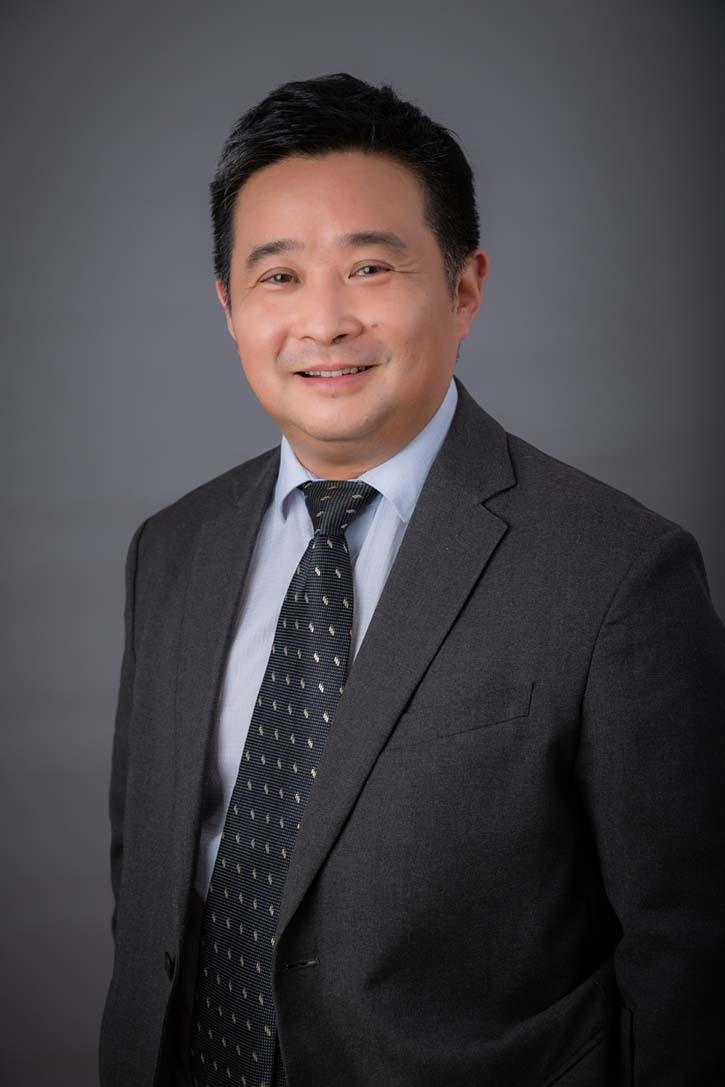# Light People: Professor Xianfeng Chen spoke about integrated photonics

**DOI:** 10.1038/s41377-022-00910-9

**Published:** 2022-07-12

**Authors:** Chenzi Guo

**Affiliations:** Light Publishing Group, Changchun Institute of Optics, Fine Mechanics and, Physics, Chinese Academy of Sciences, 3888 Dong Nan Hu Road, Changchun, 130033 China

**Keywords:** Nanophotonics and plasmonics, Photonic devices

## Abstract

In 1969, Stewart E. Miller published “Integrated optics: an introduction”, which outlined a proposal for a miniature form of laser beam circuitry, marking the first research paper about what is now known as integrated photonics. Now half a century has passed, integrated photonics grew robustly from integrating a limited number of devices and functions towards versatile and industrialized photonic integrated circuits. In this interview, *Light: Science & Applications* invited Prof. Xianfeng Chen [see the “Short Bio” section] to share his insight about the past, present and future of integrated photonics.

**Q1. How did integrated photonics change optoelectronics? What are the major challenges and opportunities for integrated photonics?**

A1: Integrated optics compresses the scale of photonic devices to the scale of electronic devices, so that both of them have the characteristics of CMOS compatibility, mutual integration, and large-scale integration, making optoelectronic integrated chips possible. At present, the applications of integrated photonics have been very wide, including optical communication, sensing, information processing, computing and optical storage. In addition to these, there are other fields such as materials science research, optical instrumentation, spectroscopy research, etc. The applications basically just enable the manipulation or detection of the intensity, phase, polarization, and spectrum. Through higher density photonic device integration and electronic integration, it is believed that optoelectronic chips will have more space in the future. Currently, for integrated optics, there is none perfect material that can cover all functions. Therefore, high-quality heterogeneous integration and low-loss and low-cost coupling loss are very important challenges.

**Q2. Silicon has long been a mature material in integrated photonics, but progresses in materials technology have brought out many new choices. Could you name a few promising materials for integrated photonics and their major strengths & weakness?**

A2: Silicon-based photonic integration platform has received great attention because of its strong potential for monolithic integration of electronics and photonics, and at the same time, it relies on the current mature low-cost, large-scale CMOS integrated circuit manufacturing process. Another good photonic integration platform is the indium phosphide material system (III-V semiconductors), which has the natural advantage of easy fabrication of light sources. However, its monolithic integration process is complex and expensive, and to achieve monolithic integration, it needs to rely on processes such as selective area growth, butt-coupling growth, or quantum well hybridization. Lithium niobate and silica waveguides have very low fiber insertion loss, so they are widely used in the optical communication industry. However, due to the limited ability of doped waveguide to confine light, it cannot be integrated on a large scale. Lithium niobate on insulator (LNOI) is a new type of thin film material that is very popular at present. It has excellent optical properties, especially large optical window, ultra-low absorption loss, very strong electro-optic effect and nonlinear effect. It has realized applications in electro-optic modulators and nonlinear frequency conversion.

**Q3. Your team has pioneered in chip-scale LiNbO**_**3**_ **-based optoelectronic devices, and after the wafer-scale, high-quality thin films of LN-on-insulator (LNOI) were commercialized, some of the thin film LN devices have already outperformed their counterparts realized in bulk LN crystals. However, challenges such as Charge Carrier Effects also came along with LN materials. Could you talk about the major challenges and solutions of LNOI optoelectronic devices?**

A3: Our team has been focusing on LN and LNOI based photonics and its applications for more than 25 years. We are one of the first research groups in China to enter this field. Shortly after the successful etching of LN, LNOI has proven its supremacy in optical manipulation by leveraging nonlinear, electro-optic, acousto-optic effects of LN. LN itself is not possible for light emission or detection. The major challenges of LNOI optoelectronics is the full integration of on-chip laser source and detectors. Feasible solution to the respective problem by ion doping and heterogeneous integration has been reported very recently. But extensive work is yet to be done.

**Figure:**
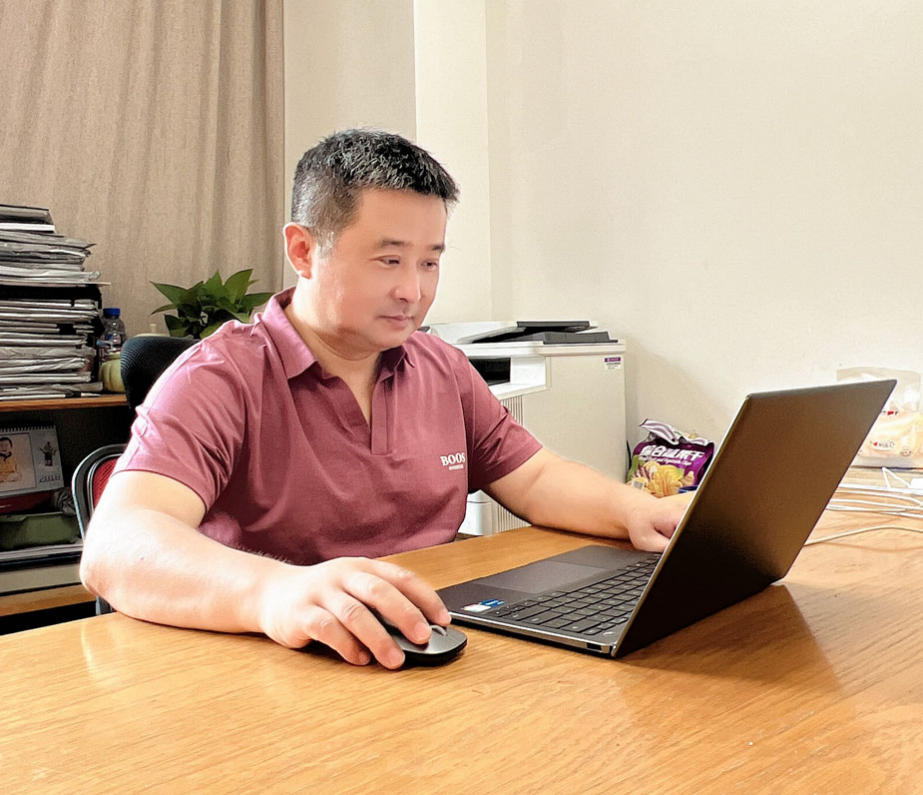
Prof. Xianfeng Chen at his office

**Figure:**
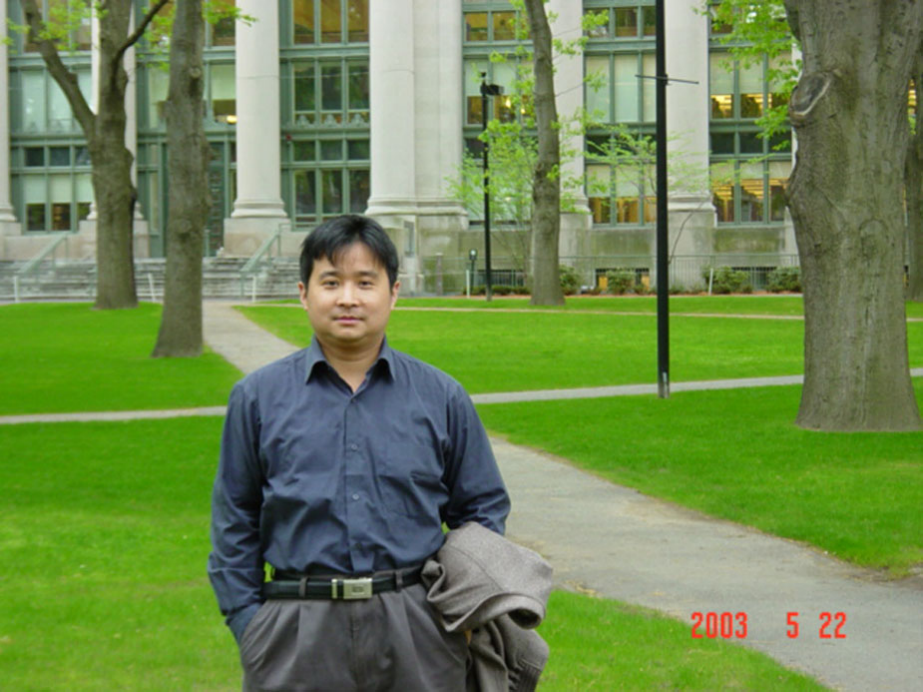
Prof. Xianfeng Chen at Harvard University with senior scholarship

**Q4. LiNbO3 is known for its large second- and third- order nonlinear susceptibility tensors, which enables efficient electro-optical modulators, nonlinear optical frequency conversion, and optical frequency combs. What changes nonlinearity dynamics (in particular in LNOI) have brought and will bring to integrated photonics? And what would be the major obstacles to overcome?**

A4: In LNOI, strong nonlinearity meets strong confinement. This would lead to ultrahigh efficient devices that are far superior to their traditional counterparts. High-performance EO modulators, frequency convertors and frequency comb generators have been demonstrated recently. They will have a significant role in communication, microwave photonics, quantum optics and other fields. For nonlinearity, it also means much stronger light-matter interaction can be triggered by using the same input power, which would provide new methods for achieving all optical processing and computing. However, this also means the system is fragile to defects. In the future, through mature large-scale manufacturing, combined with large second- and third-order nonlinearity and nanostructure properties of LN, we believe that LNOI can realize more complex nonlinear systems. Therefore, the coupling of fiber to the nano-waveguide and the power handling capability of the waveguide are the main obstacles to the application of nonlinear devices.

**Figure:**
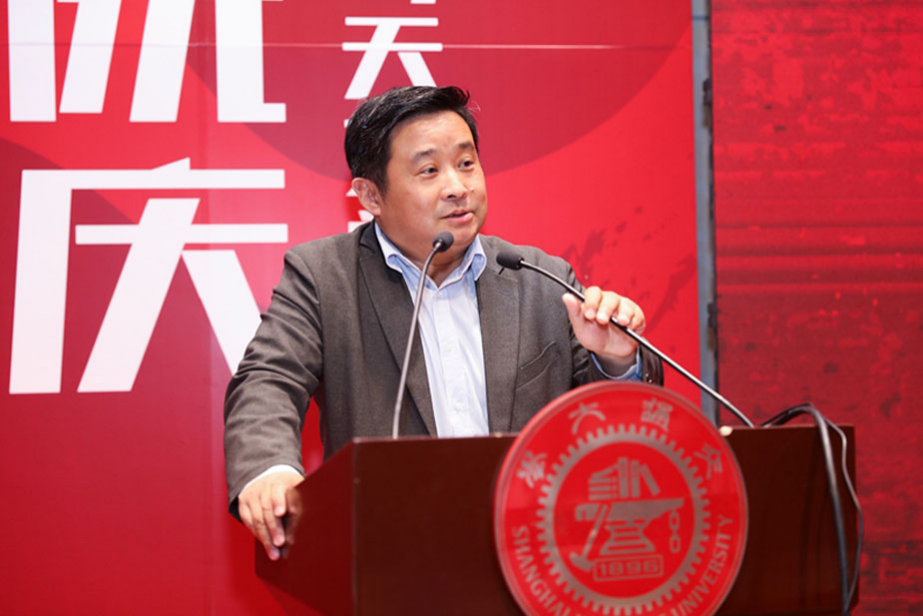
Prof. Xianfeng Chen presented talk

**Q5. What about fiber to LNOI chips coupling?**

A5: Fiber-to-chip coupling has been one of the main issues for densely integrated photonics toward applications. The problem is similar for silicon optoelectronics as well. Several coupling schemes on silicon have been directly adopted to the LNOI platform. The current paradigm for LNOI chip coupling involves mode conversion structures which mainly adopt a double-layer waveguide cone structure and the coupling loss can be reduced to less than 1 dB. Another favored scheme is grating couplers. The fabrication process of the grating coupling technology is relatively simpler, but the general coupling efficiency is not very high.

**Q6. In your recent work published in Light: Science & Applications, a periodically poled lithium niobate waveguide is introduced, and a 15-user QSDC network is experimentally demonstrated, by which any two users can communicate over 40** **km of optical fiber, with a** **>** **95% fidelity of the entangled state. What changes this work would bring? Could you predict the communication distance and transmission rate of QSDC networks in the near future? What are the major to-be-solved problems in QSDC?**

A6: Quantum secure direct communication has simpler protocols, less potential security loopholes and higher security guarantees, which continues to enhance the security and the value propositions of quantum communications in general. It is important to construct a quantum network in order to make wide applications of QSDC. In our network scheme, each user interconnects with any others through shared pairs of entangled photons at different wavelengths. With 30 ITU channels, there will be a coincidence event between each user by performing a Bell-state measurement based on sum frequency generation. This allows the four sets of encoded entangled states to be identified simultaneously without post-selection. Moreover, it is possible to improve the information transmission rate greater than 100 kbps in the case of high-performance detectors, as well as high-speed modulators. The QSDC based on entanglement network can also be used for the interconnection of multiple networks. Quantum repeaters are set up in the network structure to interconnect multiple networks in different regions. At the same time, this scheme can also be applied to experiments such as quantum teleportation.

**Q7. Could you share some perspective on LNOI for highly integrated quantum devices?**

A7: The attractive features of LNOI are the exceptional properties of LN and its capability for dense photonics integration. LN is one of the best platforms for many applications for decades, with photonics and quantum optics being the major one. However, dense integration still hasn’t been well addressed in bulk LN. With strong light confinement, light-matter interactions have reached unprecedented efficiency levels. The perspective of LNOI shows no boundary. For fundamental optical physics, the strong light-matter interaction in the nanoscale will spawn many useful devices. For highly integrated quantum devices, single photon manipulators, logic gates and quantum computing circuits are within envision.

**Q8. Moore’s law has well predicted the past years of integrated circuits, are there such well recognized laws in integrated photonics?**

A8: The development trend of integrated circuits, that is silicon electronics, has well followed Moore’s law over the past half century. However, it is difficult to directly compare integrated photonics with the mature microelectronics integration technology in terms of computing power. Because integrated photonics technology is still in its infancy. At present, the development of silicon electronics has approached the physical boundary, where the transistor has been scaled down to nanometers, a dimension of only several atomic constants. And the power consumption also gets more and more serious as transistors get more and more dense. Judging from these physical barriers, there is a consensus of “fiber optics over copper cables” in the chip. The trend would experience three stages from electronic, optoelectronic to photonic chips. Integrated photonics will gradually take over. We have every reason to expect that all photonic chips for general computing would prevail in the young generation just like silicon electronics in ours.

**Q9. You have been leading a large group with diverse research interests, and I know you have a very young and lovely kid, could you share some experiences in how to manage work life balance?**

A9: Yes, our research interests are as diverse as our group members. In my lab, we have a group of highly self-motivated young students and researchers. Everyone has his/her strength. We encourage everyone to take on the scientific topics that are both fundamentally important and fit to their own background. I would tell them, “Do what you love and love what you do.” With this, you will have all the passion to confront any difficulty. It helps to increase your motivation and engagement, thus yielding higher productivity. The whole cycle is a positive feedback loop. This is also the guideline in my work and life, which really helps me to achieve my own work-life balance. I love the scientific research that I am doing. My son is also really into science. Despite that he is only five years old, he knows a lot of basic knowledge about mathematics, astronomy, optics and even quantum physics. Science seems natural to him. For me, traditional division between work and life is fading. I am not totally leaving my work even when I am spending time with my family.

**Figure:**
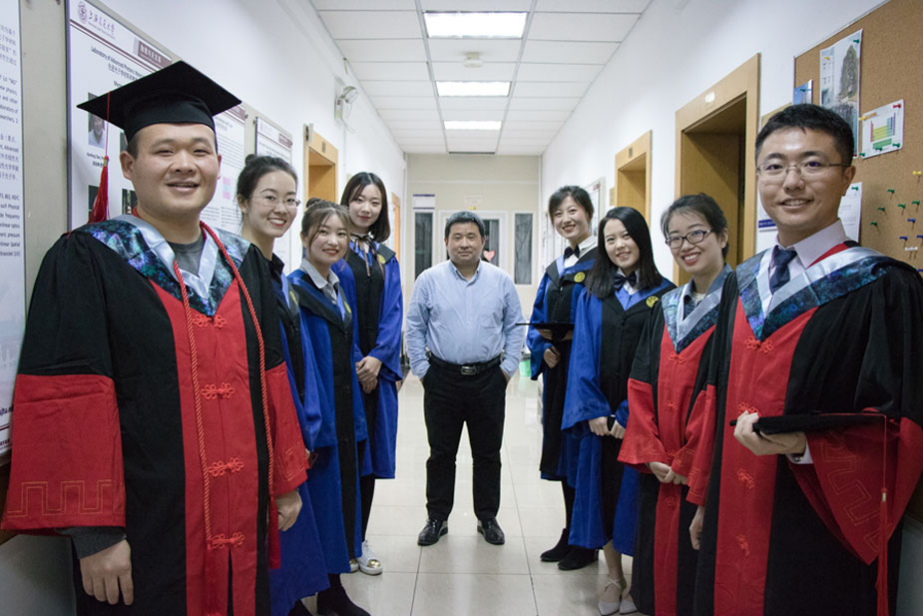
Prof. Xianfeng Chen and his group members

**Short Bio**

*Xianfeng Chen is a distinguished professor of school of physics and astronomy, Shanghai Jiao Tong University. He is the recipient of China national funds for distinguished young scientists in 2011, national high-level talent special support plan in 2015, and the grantee of the special state allowance since 2014. Now, he serves as an Editor-in-Chief of Journal of Nonlinear Optical Physics & Material (World Scientific)*.

*During the past three decade, his researches have focused on nonlinear optics, integrated optics, micro-&Nano-photonics, quantum optics, and bio-photonics. Some results have been achieved in basis theory of optical physics and its applications for national needs. Over 300 journal papers have been published in leading international refereed journals, such as Nature, Nature Photonics, Physics Review Letters, Light: Science and Applications, and so on. These papers have been cited over 5000 times. Prof. Xianfeng Chen has served as co-organizer, conference chair, section chair, and committee member of academic conferences for more than 40 times. In 2010, Prof. Xianfeng Chen won the C.N. Yang Award from AAPPS for the contribution to quasi-phase-matching nonlinear optics.*

**Figure:**